# Gender differences in infertility stress, coping and the ability to share feelings about infertility

**DOI:** 10.1186/s12978-026-02343-8

**Published:** 2026-04-23

**Authors:** Angelika Szatmári, Ildikó Kovács, János Zádori, Boglárka Pataki, Miklós Zrínyi

**Affiliations:** 1 Faculty of Health and Social Studies, University of Szeged, Temesvári krt. 31, Szeged, H-6726 Hungary; 2 Department of Psychiatry, Albert Szent-Györgyi Medical School, University of Szeged, Szeged, Hungary; 3 Institute of Reproductive Medicine, Albert Szent-Györgyi Medical School, University of Szeged, Szeged, Hungary

**Keywords:** Infertility stress, FPI, Anxiety, Stress, Depression, Coping

## Abstract

**Background:**

Infertility distress is a major concern for couples. The primary aim of this study was to predict ability to share feelings about infertility using the Fertility Problems Inventory (FPI) depression, anxiety and additional variables.

**Methods:**

Randomly selected couples responded to paper-pencil instruments that measured ability to share feelings about infertility, fertility stress, coping, stress, anxiety and depression as key variables. Selected participants were adults diagnosed with infertility undergoing assisted reproductive treatment for at least 6 months in Hungary. Multiple linear regression analysis was applied to predict fertility related stress scores separately for female and male participants.

**Results:**

A total of 94 couples participated. Employing Wilcoxon tests, a significant difference was identified in the use of emotional coping (females using more emotional coping compared to males) (z = -4.18, *p* < 0.001). No significant difference was found in the utilization of problem-solving coping. With regard to the ability to share feelings about infertility, no significant difference was identified in favor of either gender. With regard to the impact of treatment on everyday life, female respondents indicated a significantly greater effect (z = -2.67, *p* = 0.008). One-point increase in anxiety scores increased fertility distress by 0.83 and 1.25 points for women and men, respectively. One-year increase since infertility diagnosis was linked to a 1.61-point increase in fertility distress for males. For women, for every one-point increase in the perception of their inability to discuss personal issues with someone else, there was a five-fold increase in infertility-related distress.

**Conclusions:**

Stress, anxiety, and depression were linked to problem-solving in women, but were associated with emotional coping in men. Regression analysis showed a difference between women and men in predicting stress related to infertility. In women, the inability to talk about their feelings related to infertility was significantly negatively associated with fertility stress. However, in men, feelings related to infertility were not predictors of infertility stress. This result requires further research to clarify the reason for the difference.

## Abstract (plain language summary)

Infertility distress is a significant issue for couples. The main goal of this study was to predict fertility distress using the Fertility Problems Inventory (FPI).

Here’s how we did it: We asked 94 couples to answer questions on a questionnaire about their coping strategies, stress levels, anxiety levels, and depression levels.

Here are the results: It turned out that women used more emotional coping strategies than men. Female respondents said that the treatment had a much bigger impact on their everyday life. For both women and men, higher anxiety was linked to more distress related to fertility. Each one-point increase in anxiety scores led to an 0.83-point increase in distress for women and a 1.25-point increase for men. Additionally, each year since being diagnosed with infertility was associated with a 1.61-point increase in distress for men. For women, every one-point increase in the feeling that they could not talk about personal issues with someone else was linked to a fivefold increase in infertility-related distress.

In light of these findings, we can draw the following conclusions: Women who were stressed, anxious, or depressed tended to use problem-solving strategies, while men were more likely to rely on emotional coping. However, the study did not find a significant link between coping strategies, depression, stress, and infertility distress.

Finally, among women, an inability to discuss feelings related to infertility was associated with higher levels of fertility stress. However, in men, feelings related to infertility did not predict infertility distress.

## Background

Infertility, defined as the inability to conceive after 12 months of unprotected intercourse [1], is a significant global health problem affecting millions of couples. According to the report of the Guttmacher–Lancet Commission, a total of 49 million to 180 million couples might globally experience infertility [2]. Despite advancements in treatment, infertility prevalence has not decreased [3]; [4]. While often perceived as a female issue, male infertility accounts for 20–30% of cases and is a factor in 50% of unsuccessful fertility attempts [5].

The psychological impact of infertility is profound, leading to increased anxiety, depression, and relationship strain, with some couples experiencing “emotional divorce” [6–13]. This emotional toll is not distributed equally between genders. Women frequently exhibit higher levels of distress, depression, and anxiety compared to men [14–17]. Women also tend to employ maladaptive coping strategies, such as passive coping [18], venting, and self-blame [19], which can worsen anxiety and depression [20]. As with women, the coping strategies employed by men in their dyadic relationships are of equal importance to marital well-being [21]; [22].

Infertility stress significantly impairs quality of life and sexual satisfaction for both partners [23–27]. However, studies show gender-specific differences in how coping mechanisms relate to mental health outcomes. For instance, problem-solving is linked to better psychological health and quality of life for men, while the positive effects of all coping strategies diminish over time for both genders, particularly after three years of infertility [17].

This body of research underscores the critical role of stress and coping in managing infertility. While most studies confirm gender-based differences in coping and adjustment, they often use the same measures, like the Fertility Problem Inventory (FPI), without creating separate predictive models for men and women [28]; [29]. Therefore, this study aimed to predict the capacity of participants to share feelings about their infertility experiences (which is a critical issue with regard to quality relationship adjustment) using stress, coping, anxiety, and other variables to develop individual regression models for women and men.

## Methods

The data presented in this study were collected from couples undergoing infertility treatment at the Institute of Reproductive Medicine, University of Szeged, Hungary, between May 2022 and November 2022.

- Inclusion criteria were as follows: a confirmed diagnosis of infertility and participation in infertility treatment at the same clinical center prior to study enrollment and a minimum of 18 years of age. The inclusion criteria necessitated that couples partake in assisted reproduction a minimum of six months prior to the commencement of the study (IVF, ICSI, etc.). Our target group was women and men with reproductive disorders undergoing assisted reproductive interventions.
- Exclusion criteria included: any diagnosed psychiatric or neurological disorder. During the initial recruitment phase, a total of 120 test packs were distributed.

Of these, 94 completed questionnaires were returned, resulting in a final sample of 188 subjects. The sampling procedure involved the random recruitment of couples with reproductive disorders who presented to the reproductive clinic, met the inclusion criteria, and underwent assisted reproductive interventions.

The completion of the aforementioned questionnaires was required to be done in printed form. Patients were requested to complete questionnaires at the time of the study’s initiation and after a six-month follow-up period. In order to minimize interdependence of responses, a separate room was provided for couples who had to complete the survey while staying at the clinic. There was no time limit for completion. Although clinical staff assisted in enrolling patients in the study, patient recruitment was conducted by a member of the research team. Patients were assured that participation in the study would not affect their treatment.

The completed questionnaires were to be returned in a sealed envelope to the research staff. The clinical staff entered a code number on the envelopes, which was generated in advance by the research team. It was not possible to identify the respondents afterwards. The consent forms from the research subjects were collected by the research staff in another sealed envelope, separate from the questionnaires. Both envelopes were handed personally to the principal investigator. A member of the research team was available, either in person or on call, to answer questions or provide clarification if participants needed assistance.

Data entry was based on unique identifiers assigned to couples and recorded on envelopes. The research assistant managing the process had no access to patient identification and personal information. Both parties of the couple had to complete the survey for it to be included in the final analysis.

### Ethics

The study was conducted in accordance with the tenets of the Declaration of Helsinki and was approved by the Ethics Committee for Human Institutional and Regional Biomedical Research of the University of Szeged (Ethics approval number: 8/2021-SZTE RKEB). Written informed consent was obtained from all participants prior to data collection. All participants who decided to take part in the research completed the study.

### Sample size calculations

A priori sample calculation was conducted for multiple regression analysis using the GPower software (GPower, 2024). The calculation indicated that, assuming a medium effect size of 0.15, a significance level of 5% (0.05), statistical power at 0.80 (20% probability of type II error), and eight independent variables as predictors, a total sample size of 109 subjects was required. In other words, the minimum number of subjects required for each group (separately for female and male regression models) was 109. The final sample size of 94 subjects per group yielded a statistical power of 0.73 in the post hoc analysis. This was one such study limitation that prevented immediate acceptance and generalizability of the results obtained.

### Statistical analysis

Descriptive statistics were employed to present the characteristics of the sample. One-sample Kolmogorov-Smirnov tests were employed to ascertain the normal distribution of the primary measures. Given the non-normal distributions observed, nonparametric tests were employed when appropriate. Spearman correlation coefficients were employed for the purpose of evaluating potential associations. To assess the differences between couples, Wilcoxon signed-rank tests were performed. A series of multiple linear regression models were constructed in order to predict an individual’s capacity to engage in discussions pertaining to their feelings about fertility, as well as to predict their FPI scores. Outliers, defined as cases with standardized residual values greater than +/- 2.0, were excluded from subsequent analyses. No attempt was made to replace missing data. All analyses were conducted using IBM SPSS Statistics for Windows, version 29.0.

### Instruments

First, participants responded to a self-administered survey instrument that included sociodemographic and infertility questions such as age, gender, education, marital status, employment status, type of work, place of residence, family income, length of time trying to conceive, duration of infertility, and cause of infertility.

Additional, validated research tools were also used. The *Perceived Stress Scale (PSS)* was used to measure psychological distress [30]. It is a 14-item, self-report questionnaire designed to measure the degree to which situations in one’s life are perceived as stressful. The PSS measures how unpredictable, uncontrollable, and overwhelming respondents find their lives. The scale also includes a series of direct questions about current levels of experienced stress. Responses are recorded on a 4-point Likert scale (0 = never, 4 = very often). Higher scores on the scale indicate greater stress. A reliability of 0.89, measured as Cronbach’s alpha, was achieved in this study.

The assessment of depressive symptoms was conducted using the *Beck Depression Inventory Short-Form (BDI-S)*. The scale comprises nine items that assess common symptoms of depression, including pessimism, dissatisfaction, guilt, social introversion, indecisiveness, work inhibition, insomnia, fatigue, and somatic anxiety [31]. Responses are rated on a four-point Likert scale, with values ranging from 0 (not frequent) to 3 (very frequent). Higher scores on the BDI-S indicate a higher degree of depressive symptoms. A reliability of 0.847 was demonstrated in this research.

Anxiety levels were evaluated using the *Spielberger State-Trait Anxiety Inventory (STAI)* trait subscale [32]; [33]. The STAI-T is comprised of 7 reversed (negative) and 13 positive items that assess the general tendency of anxiety. It is rated on a 4-point Likert scale, ranging from 1 (not at all) to 4 (very much). Higher scores indicate elevated levels of anxiety. The instrument demonstrated a reliability coefficient of 0.925 in the present study.

We used the Hungarian 22-item version of the Ways of Coping Questionnaire (WCQ), originally developed by Lazarus and Folkman, to assess the coping strategies of couples experiencing infertility [34, 35]. We chose the 22-item version because it was validated on the largest Hungarian sample (20,902 general population members) [34]. The 16-item version was tested on a smaller group (12,634), and the 14-item version (validated on high school students and healthcare professionals) was deemed unsuitable for this population [34, 36]. 

The 22 items measure problem-focused and emotion-focused coping, grouped into seven subscales:

- Goal-oriented action
- Problem analysis
- Adaptation
- Emotional action
- Seeking emotional balance
- Withdrawal
- Seeking peer support

Responses were recorded on a four-point Likert scale (0 = not common to 3 = very common). Problem-solving coping score was calculated as the sum of items 1, 8, 9, 17, 18, 21, and 22 on the seven subscales. Emotional coping score was the sum of the remaining 15 items. The full-scale reliability coefficient (Cronbach’s alfa) in our study was 0.716.

Finally, the level of distress associated with infertility was assessed using the *Fertility Problems Inventory (FPI-9)* [37]. The questionnaire comprises 46 items, which are scored on a 6-point Likert scale ranging from 1 (strongly agree) to 6 (strongly disagree). The instrument is divided into five subscales: (1) social anxiety; (2) sexual anxiety; (3) relationship anxiety; (4) rejection of a child-free lifestyle; and (5) need for parenthood. Higher scores on each scale indicate increased levels of distress related to infertility. The scale exhibited a reliability coefficient of 0.854 during the course of this research.

Furthermore, participants were requested to respond to two additional items on a 4-point Likert scale, namely, ‘To what extent does infertility treatment impact your daily life? (1 = not at all, 4 = very much)’ and ‘I do not feel comfortable sharing my feelings with anyone regarding my problems (1 = strongly disagree, 4 = strongly agree).’

## Results

### Demographics

A total of 94 couples, defined as female/male partners, completed the study. The mean age of the female participants was 34.9 years (SD = 0.49), while the male participants reported an average age of 38.9 years (SD = 0.60). The reason for the infertility was unclear, as it might have been present in both partners. Because diagnosis was established at different times, it took females an average of 34.5 months (SD 4.45) to receive an infertility diagnosis, while males reported of 25.7 months (SD 3.56). With regard to the length of time (in months) spent trying to have children, females reported a mean of 46.8 (SD 3.03), while males reported a mean of 48.3 (SD 2.93). This minimal difference between the two sexes can probably be explained by their respective recollection abilities. Table 1 presents a descriptive analysis of the main study variables, stratified by gender. One sample Kolmogorov-Smirnov tests, because of significant results, confirmed non-normal distribution of main study measures.

**Table 1:** Descriptive Statistics by Gender

Gender				Statistic	Std. Error
How does treatment influence your everyday life?	Male	Mean		1.46	0.079
		95% Confidence Interval for Mean	Lower Bound	1.31	
			Upper Bound	1.62	
		Std. Deviation		0.655	
		Minimum		1	
		Maximum		4	
	Female	Mean		1.81	0.098
		95% Confidence Interval for Mean	Lower Bound	1.62	
			Upper Bound	2.01	
		Std. Deviation		0.873	
		Minimum		1	
		Maximum		4	
I don't feel I am able to share my feelings with anyone	Male	Mean		1.97	0.124
		95% Confidence Interval for Mean	Lower Bound	1.72	
			Upper Bound	2.22	
		Std. Deviation		1.029	
		Minimum		1	
		Maximum		4	
	Female	Mean		1.83	0.108
		95% Confidence Interval for Mean	Lower Bound	1.61	
			Upper Bound	2.04	
		Std. Deviation		0.965	
		Minimum		1	
		Maximum		4	
FPI (Fertility Problems Inventory)	Male	Mean		183.45	2.677
		95% Confidence Interval for Mean	Lower Bound	178.11	
			Upper Bound	188.79	
		Std. Deviation		22.240	
		Minimum		116	
		Maximum		228	
	Female	Mean		171.86	2.983
		95% Confidence Interval for Mean	Lower Bound	165.93	
			Upper Bound	177.80	
		Std. Deviation		26.679	
		Minimum		103	
		Maximum		231	
Problem solving coping	Male	Mean		11.75	0.408
		95% Confidence Interval for Mean	Lower Bound	10.94	
			Upper Bound	12.57	
		Std. Deviation		3.389	
		Minimum		3	
		Maximum		21	
	Female	Mean		12.38	0.394
		95% Confidence Interval for Mean	Lower Bound	11.59	
			Upper Bound	13.16	
		Std. Deviation		3.527	
		Minimum		3	
		Maximum		20	
Emotional coping	Male	Mean		16.12	0.647
		95% Confidence Interval for Mean	Lower Bound	14.83	
			Upper Bound	17.41	
		Std. Deviation		5.373	
		Minimum		4	
		Maximum		28	
	Female	Mean		18.85	0.631
		95% Confidence Interval for Mean	Lower Bound	17.59	
			Upper Bound	20.11	
		Std. Deviation		5.648	
		Minimum		4	
		Maximum		38	
BDI-S (Beck Depression Inventory)	Male	Mean		3.33	0.458
		95% Confidence Interval for Mean	Lower Bound	2.42	
			Upper Bound	4.25	
		Std. Deviation		3.803	
		Minimum		0	
		Maximum		16	
	Female	Mean		4.44	0.535
		95% Confidence Interval for Mean	Lower Bound	3.37	
			Upper Bound	5.50	
		Std. Deviation		4.784	
		Minimum		0	
		Maximum		19	
STAIT (State Trait Anxiety)	Male	Mean		39.28	1.265
		95% Confidence Interval for Mean	Lower Bound	36.75	
			Upper Bound	41.80	
		Std. Deviation		10.512	
		Minimum		23	
		Maximum		65	
	Female	Mean		42.54	1.278
		95% Confidence Interval for Mean	Lower Bound	39.99	
			Upper Bound	45.08	
		Std. Deviation		11.429	
		Minimum		23	
		Maximum		69	
PSS (Perceived Stress Scale)	Male	Mean		23.09	0.874
		95% Confidence Interval for Mean	Lower Bound	21.34	
			Upper Bound	24.83	
		Std. Deviation		7.261	
		Minimum		6	
		Maximum		41	
	Female	Mean		24.11	0.970
		95% Confidence Interval for Mean	Lower Bound	22.18	
			Upper Bound	26.04	
		Std. Deviation		8.676	
		Minimum		7	
		Maximum		48	
Family planning (yrs)	Male	Mean		3.8273	0.24784
		95% Confidence Interval for Mean	Lower Bound	3.3327	
			Upper Bound	4.3218	
		Std. Deviation		2.05869	
		Minimum		1.50	
		Maximum		11.00	
	Female	Mean		4.0510	0.28416
		95% Confidence Interval for Mean	Lower Bound	3.4854	
			Upper Bound	4.6166	
		Std. Deviation		2.54158	
		Minimum		0.08	
		Maximum		15.00	
Infertility problems (yrs)	Male	Mean		2.1425	0.33867
		95% Confidence Interval for Mean	Lower Bound	1.4667	
			Upper Bound	2.8183	
		Std. Deviation		2.81323	
		Minimum		0.00	
		Maximum		15.00	
	Female	Mean		3.1240	0.42028
		95% Confidence Interval for Mean	Lower Bound	2.2874	
			Upper Bound	3.9605	
		Std. Deviation		3.75910	
		Minimum		0.00	
		Maximum		24.00	

Table 2 presents the Spearman correlation coefficients between coping strategies by gender and the main variables.

**Table 2:** Correlations by Gender and Coping Strategy

		Problem solving coping_females	FPI	BDI-S	PSS	STAIT	Treatment (yrs)	Infertility (yrs)
Problem solving coping_females	Correlation Coefficient	1.000	0.135	-.356^**^	-.426^**^	-.383^**^	-0.083	-0.110
	Sig. (1-tailed)		0.102	< 0.001	< 0.001	< 0.001	0.213	0.145
	N	94	90	94	89	91	94	94
		Problem solving coping_males	FPI	BDI-S	PSS	STAIT	Treatment (yrs)	Infertility (yrs)
Problem solving coping_males	Correlation Coefficient	1.000	0.103	-0.077	-0.171	-.245^*^	-0.053	-0.102
	Sig. (1-tailed)		0.175	0.235	0.056	0.012	0.309	0.168
	N	91	85	90	88	84	91	91
Emotional coping_females	Correlation Coefficient	Emotional coping_females	FPI	BDI-S	PSS	STAIT	Treatment (yrs)	Infertility (yrs)
		1.000	-0.131	0.154	0.032	0.146	0.023	-.226^*^
	Sig. (1-tailed)		0.110	0.070	0.384	0.085	0.412	0.015
	N	93	89	93	88	90	93	93
Emotional coping_males	Correlation Coefficient	Emotional coping_males	FPI	BDI-S	PSS	STAIT	Treatment (yrs)	Infertility (yrs)
		1.000	-0.146	.314^**^	.261^**^	.326^**^	.180^*^	0.024
	Sig. (1-tailed)		0.093	0.001	0.007	0.001	0.045	0.410
	N	90	84	89	87	84	90	90

*FPI * Fertility Inventory,  *BDI-S* Beck Depression Inventory, *PSS* Perceived Stress Scale,  *STAIT* State Trait Anxiety

### Coping

The utilization of a problem-solving coping strategy was inversely correlated with the prevalence of depressive, stress, and anxiety symptoms among female participants. The utilization of problem-solving coping strategies was found to be inversely associated with elevated levels of depression, stress, and anxiety among women. With regard to the male participants, anxiety was the sole variable that exerted a negative influence on the utilization of problem-solving coping strategies. Specifically, elevated levels of anxiety were associated with a reduction in the deployment of problem-solving coping strategies.

With regard to the utilization of emotional coping strategies, the infertility diagnosis emerged as the sole variable that exerted a negative influence on emotional coping among female respondents, with the duration of treatment exhibiting an inverse relationship.

However, the male participants demonstrated a positive correlation between depression, stress, anxiety, and family planning (i.e., treatment) and the utilization of an emotional coping style. Specifically, an increase in depression, stress, anxiety, and treatment duration was associated with a higher frequency of emotional coping.

Table 3 illustrates the results of the Wilcoxon test, which examined the difference between the frequency of problem-solving and emotional coping strategies utilized by female and male couples. A significant difference was identified in the use of emotional coping between couples (females using more emotional coping compared to males) (z = -4.18, *p* < 0.001). Nevertheless, no significant difference was identified in the utilization of problem-solving coping.

**Table 3:** Wilcoxon-Sign Test for Differences in Coping by Gender

	N	Mean	Std. Deviation	Minimum	Maximum
Emotional coping females	93	19.26	5.739	4	38
Emotional coping males	90	16.24	5.399	4	28
Problem solving coping females	94	12.60	3.693	3	20
Problem solving coping males	91	11.99	3.767	3	21

Ranks					
		N	Mean Rank	Sum of Ranks	
Emotional coping males - Emotional coping females	Negative Ranks	58	41.99	2435.50	
	Positive Ranks	21	34.50	724.50	
	Ties	10			
	Total	89			
Problem solving males - Problem solving females	Negative Ranks	45	37.50	1687.50	
	Positive Ranks	29	37.50	1087.50	
	Ties	17			
	Total	91			

Test Statistics^a^					
	Emotional coping males - Emotional coping females	Problem solving coping males - Problem solving coping females			
Z	-4.186^b^	-1.621^b^			
Asymp. Sig. (2-tailed)	< 0.001	0.105			

a. Wilcoxon Signed Ranks Test

b. Based on positive ranks

### Impact on daily living

A comparable analysis was conducted to ascertain the discrepancy between couples in terms of the impact of treatment on their daily lives and the extent to which they felt unable to discuss their feelings regarding their infertility issues. With regard to the ability to share feelings about infertility, no significant difference was identified in favor of either gender. Table 4 presents the Wilcoxon-Sign tests for differences in feelings by gender.

**Table 4:** Wilcoxon-Sign Tests for Differences in Feelings by Gender

	N	Mean	Std. Deviation	Minimum	Maximum
How much does treatment affect your life females	93	1.7849	0.83209	1.00	4.00
How much does treatment affect your life males	91	1.4945	0.63899	1.00	4.00
I don't feel I am able to share my feelings with anyone females	93	1.8065	0.95846	1.00	4.00
I don't feel I am able to share my feelings with anyone males	88	1.9432	0.99837	1.00	4.00

Ranks					
		N	Mean Rank	Sum of Ranks	
Affect life male - Affect life female	Negative Ranks	31	23.87	740.00	
	Positive Ranks	14	21.07	295.00	
	Ties	46			
	Total	91			
Share feelings male - Share feelings female	Negative Ranks	14	10.50	147.00	
	Positive Ranks	16	19.88	318.00	
	Ties	58			
	Total	88			

Test Statistics^a^					
	Affect life male - Affect life female	Share feelings male - Share feelings female			
Z	-2.670^b^	-1.805^c^			
Asymp. Sig. (2-tailed)	0.008	0.071			

a. Wilcoxon Signed Ranks Test

b. Based on positive ranks

c. Based on negative ranks

With regard to the impact of treatment on everyday life, female respondents indicated a significantly greater effect (z = -2.67, *p* = 0.008) compared to their partners.

### Capacity to share feelings about infertility

Two multiple linear regression models have been developed to predict the capacity to share feelings about the infertility problem by gender (see Table 5) and to predict Fertility Problem Inventory scores (see Table 6). Outliers falling outside the ± 2.0 range obtained by standardized residuals were excluded from the subsequent analyses. The models were found to be significant (F_feelings_ = 4.63, *p* < 0.001 and F_FPI_ = 13.58, *p* < 0.001 – females, and F_feelings_ = 4.46, *p* < 0.001 and F_FPI_ = 4.47, *p* < 0.001 – males), with an explanatory power of 35.3% (females) and 38.5% (males) in predicting the ability to share feelings about infertility. With regard to the prediction of FPI scores, the model demonstrated an explanatory power of 61.5% (R2 = 0.615) for women and 38.6% (R2 = 0.386) for men.

**Table 5:** Regression Model Predicting Being Able to Share Feelings

Model for Females		Unstandardized Coefficients		Standardized Coefficients	t	Sig.	Collinearity Statistics	
		B	Std. Error	Beta			Tolerance	VIF
1	(Constant)	3.945	1.164		3.390	0.001		
	FPI	-0.010	0.004	-0.325	-2.393	0.019	0.516	1.939
	Problem solving coping	0.001	0.029	0.004	0.036	0.971	0.629	1.590
	Emotional coping	-0.032	0.017	-0.216	-1.846	0.069	0.696	1.436
	BDIR	0.074	0.030	0.424	2.433	0.018	0.313	3.195
	STAIT	-0.006	0.016	-0.083	-0.387	0.700	0.207	4.822
	PSS	0.000	0.017	0.002	0.010	0.992	0.304	3.289
	Treatment (yrs)	-0.004	0.037	-0.013	-0.119	0.906	0.805	1.243
	Infertility diagnosis (yrs)	0.012	0.024	0.056	0.522	0.604	0.833	1.201

Model for Males		Unstandardized Coefficients		Standardized Coefficients	t	Sig.	Collinearity Statistics	
		B	Std. Error	Beta			Tolerance	VIF
1	(Constant)	3.561	1.344		2.650	0.010		
	FPI	-0.007	0.005	-0.152	-1.281	0.205	0.769	1.301
	Problem solving coping	-0.037	0.036	-0.130	-1.034	0.306	0.686	1.458
	Emotional coping	-0.008	0.024	-0.044	-0.341	0.734	0.640	1.563
	BDIR	0.160	0.043	0.609	3.680	0.001	0.395	2.534
	STAIT	-0.026	0.018	-0.273	-1.459	0.150	0.308	3.246
	PSS	0.004	0.023	0.031	0.184	0.855	0.375	2.666
	Treatment (yrs)	0.110	0.052	0.236	2.099	0.040	0.855	1.169
	Infertility diagnosis (yrs)	0.034	0.039	0.101	0.869	0.389	0.805	1.242

Dependent Variable: I don't feel I can share my feelings with anyone

**Table 6:** Regression Predicting Fertility Problems Inventory Scores

Model for Females		Unstandardized Coefficients		Standardized Coefficients	t	Sig.	Collinearity Statistics	
		B	Std. Error	Beta			Tolerance	VIF
1	(Constant)	243.500	15.643		15.566	0.000		
	Problem solving coping	-0.318	0.668	-0.044	-0.476	0.636	0.658	1.520
	Emotional coping	-0.688	0.396	-0.157	-1.739	0.087	0.693	1.442
	BDI-S (depression)	-1.208	0.686	-0.230	-1.760	0.083	0.331	3.022
	STAIT (state trait anxiety)	-0.830	0.357	-0.368	-2.323	0.023	0.225	4.443
	PSS (stress)	-0.310	0.396	-0.105	-0.783	0.437	0.315	3.175
	Treatment (yrs)	0.849	0.816	0.086	1.041	0.302	0.826	1.211
	Infertility diagnosis (yrs)	0.213	0.546	0.032	0.389	0.698	0.828	1.208
	I don't feel I am able to share my feelings with anyone about infertility	-5.073	2.206	-0.192	-2.299	0.025	0.810	1.235

Model for Males		Unstandardized Coefficients		Standardized Coefficients	t	Sig.	Collinearity Statistics	
		B	Std. Error	Beta			Tolerance	VIF
1	(Constant)	231.487	14.893		15.543	0.000		
	Problem solving coping	-0.355	0.724	-0.059	-0.490	0.626	0.742	1.348
	Emotional coping	0.122	0.472	0.032	0.259	0.797	0.686	1.459
	BDI-S (depression)	0.261	0.933	0.049	0.280	0.780	0.356	2.808
	STAIT (state trait anxiety)	-1.255	0.359	-0.637	-3.497	0.001	0.325	3.075
	PSS (stress)	0.191	0.460	0.068	0.414	0.680	0.400	2.499
	Treatment (yrs)	1.482	1.096	0.152	1.352	0.182	0.848	1.179
	Infertility diagnosis (yrs)	-1.616	0.808	-0.227	-2.000	0.050	0.839	1.192
	I don't feel I am able to share my feelings with anyone about infertility	-2.150	2.343	-0.110	-0.918	0.363	0.744	1.345

a. Dependent Variable: Fertility Problem Inventory

Table 5 indicates that Fertility Problem Inventory (FPI) scores and depression significantly predicted the capacity to share feelings about infertility for females, and depression and the duration of treatment for males. A one-point increase on the FPI scale was associated with a statistically significant reduction in the score on the ability to share feelings scale by 0.01 points. Because of scoring on the FPI scale, this suggested that female respondents who experienced higher levels of infertility-related distress perceived themselves as more able to express their feelings about their infertility. Conversely, a one-point increase on the depression scale was associated with a statistically significant increase in the score on the ability to share feelings scale by 0.074 points. This indicates that respondents who experienced higher levels of depressive symptoms perceived themselves as less able to discuss their feelings.

A one-point increase in depression scores for males was associated with a 0.16-point reduction in their ability to share feelings about infertility, a finding that was consistent with the scoring direction on the FPI scale. An increase of one year in the duration of treatment was associated with a reduction in the ability to discuss feelings among males of 0.11 points.

Table 6 presents the results of the regression analyses predicting the FPI scores for both female and male partners. In the female model, anxiety was the only significant variable. A one-point increase in anxiety scores was associated with a 0.83-point reduction in FPI scores. However, due to the scoring direction on the FPI scale, this resulted in an actual increase of 0.83 points in fertility-related problem perceptions. With regard to male partners, a one-point increase in anxiety was associated with a 1.25-point reduction in FPI scores, indicating a worsening of fertility-related problem perceptions. Similarly, a one-year increase since an infertility diagnosis was linked to a 1.61-point decline in FPI scores, suggesting an intensification of these perceptions.

## Discussion

The study examined factors predicting fertility attitudes and behaviors among infertile couples using the Fertility Problems Inventory (FPI) [37]. Women reported higher stress, depression, and anxiety than men, consistent with prior research [6]; [8]; [11]. Gender differences were pronounced: women reported stronger negative impacts of infertility treatment, while men more often felt unable to share their feelings. 

Coping styles also differed. Both sexes used problem-focused coping similarly, but women relied more on emotional coping, linked to poorer psychological outcomes [18]. For women, greater use of problem-focused coping correlated with lower anxiety, depression, and stress, whereas for men it correlated only with reduced anxiety. Women’s emotional coping declined with longer infertility duration, while men’s emotional coping increased with stress, anxiety, depression, and treatment length. Thus, stress, anxiety, and depression shaped problem-solving in women but emotional coping in men, suggesting interventions should strengthen problem-solving in women and reduce emotional coping in men. The latter is also important because Zurlo [17] pointed out that the use of problem-solving coping resulted in significant positive changes in quality of life primarily among men. Surprisingly, FPI scores showed no correlation with coping strategies. This finding may not correspond to research outcomes reported earlier [14–17]. We cannot yet explain why we did not find a correlation in this study. Further research is needed to understand whether this is a characteristic of the Hungarian population or a previously unreported feature of the measurement tools used.

Regression analysis highlighted gender-specific predictors of the item “I don’t feel I can share my feelings with anyone.” For women, higher FPI scores decreased this perception, while depression increased it. For men, depression and treatment duration both increased it. Reducing depression appears critical to improving openness to be able to discuss feelings about infertility in both sexes. We did not find any detailed analysis of this issue in preliminary research; therefore, we are not in a position to compare our results at this point.

Separate regression models for FPI scores showed anxiety as a key factor for both sexes, but with stronger effects in men. This result differs from international literature, where women’s anxiety levels are typically estimated to be higher than those of men [16]; [17]. We do not have a specific explanation for this discrepancy at present, but we presume that it may be a characteristic of relationship dynamics or a result of the sample selection. In women, inability to share feelings had the greatest impact, while in men, longer infertility duration predicted worse scores. Interventions should therefore target reducing anxiety in both sexes but additionally focus on enhancing women’s ability to share more of their lived experiences related to infertility.

## Conclusions

The research found that stress, anxiety, and depression affected both men and women dealing with infertility. However, women used problem-solving coping mechanisms, while men used emotional coping more often. Despite shared variables, an intervention to improve problem-solving must account for these gender differences. The study found a link between infertility distress and anxiety in both genders, especially in men.

Regression results showed that coping mechanisms did not significantly predict infertility distress. This finding doesn’t negate the importance of coping, but suggests it may be a less important factor in predicting infertility distress scores.

A key finding for women was that a perceived inability to discuss personal issues increased infertility distress fivefold. This suggests that a crucial intervention for women may be helping them openly discuss their concerns.

In conclusion, men and women share common emotional responses to infertility distress, such as anxiety, but also have gender-specific factors, like the ability to discuss their concerns. Before accepting study findings that coping mechanisms were not a significant factor, we recommend conducting research on a larger and more representative population.

### Limitations

The authors recognize that the study sample was drawn from a single institution, which constrains the generalizability of the findings and their applicability to other samples. The sample size, which was below the level initially estimated, diminished the statistical power, which in turn affected the conclusions that could be drawn. The authors believe that repeating the study is essential to establish the findings as a basis for any future clinical intervention.

## Data Availability

While the instruments and information utilized in the data gathering process are predominantly accessible in Hungarian, the researchers offer the data to those who submit a funded proposal.
